# Examination of the interrelationships between nutrition, environmental sustainability and food-processing: A concept study using model diets

**DOI:** 10.1016/j.crfs.2023.100627

**Published:** 2023-11-04

**Authors:** Steven L. Mulrooney, James G. Lyng, Cathal O'Hara, Aifric O'Sullivan, E. Dolores O'Riordan, Eileen R. Gibney

**Affiliations:** Institute of Food and Health, University College Dublin, Ireland

## Abstract

Recent work has focused on understanding the link between diet quality and environmental impact, however it is also important to consider the role food processing plays in this relationship. Using model meal plans, this paper examines the link between nutrient content, environmental impact, and processing. Four distinct meal plans were considered – ‘Healthy’, ‘Unhealthy’, ‘Healthy (plant-based)’, ‘Healthy (plant-based, processed)’. For each a variety of environmental impact, processing and nutritional composition metrics were compared. Alternative healthy eating index (AHEI) score for the Unhealthy diet was significantly lower than the other three diets. The ‘Healthy (plant-based)’ diet had the highest AHEI score but was not significantly different to the ‘Healthy (plant-based, processed)’ and ‘Healthy’ diet scores. The greenhouse gas emissions for the two plant based diets were not significantly different to each other or to the ‘Healthy’ diet but were significantly lower than the ‘Unhealthy’ diet. The ‘Healthy’, ‘Unhealthy’, and ‘Healthy (plant-based)’ diets had similar processing specific energy consumption values however, the ‘Healthy (plant-based, processed)’ diets had significantly greater specific energy consumption. There was no clear link between diet quality and food processing when considered using processing specific energy value. When the number of processes in each diet was estimated, the unhealthier diet had considerably more processes associated with it. Examining the interaction of nutritional quality, environmental impact and processing of diets in this way highlights the complexity of the inter-relationships. Understanding these interactions is necessary to support the transition to healthy diets from sustainable sources.

## Highlights

• Consumers want convenient, healthy, sustainable foods.
• Examined link between diet-quality, environmental impact, and food processing.
• No clear link between diet quality and food processing.
• No clear link between environmental impact and food processing.
• Highlights the complexity of the healthy sustainable food system.

## Graphical abstract

**Figure:**
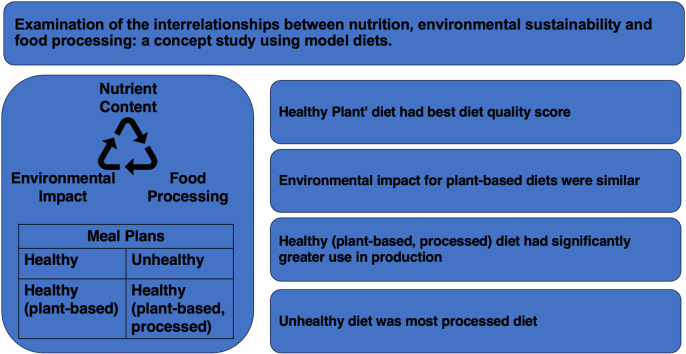

## 1 Introduction

Diet quality is considered a key factor in preventing non-communicable diseases (Grosso and Di Cesare, 2021; Branca et al., 2019). There are clear and known associations between nutrient intakes and health; for example optimal folic acid intake reduces risk of neural tube defects (Greenberg et al., 2011), calcium and vitamin D maintain bone health (Capozzi et al., 2020). Beyond nutrients, there is also interest in dietary patterns, which characterise meals and foods commonly consumed, including Mediterranean and DASH diets both known to reduce the risk of non-communicable diseases including cardiovascular disease or hypertension (Schulze et al., 2018).

In recent decades consumer food choices have evolved, with a shift towards convenience and variety in the diet, and more recently an increase in the demand for sustainable foods and ingredients (Harris and Shiptsova, 2007; Mertens et al., 2016; Willett et al., 2019). To fulfil these consumer expectations, the food industry has developed a wide range of convenience foods, using various food processing techniques (Floros et al., 2010). Food processing has existed for thousands of years and encompasses techniques to ensure food safety, to preserve, alter the properties of the food to add value or to enhance convenience (Huebbe and Rimbach, 2020). Processes including size reduction (e.g. cutting), drying, cooking, cooling and brining were discovered thousands of years ago and their use in the modern food chain have been refined as food technology has advanced (Huebbe and Rimbach, 2020). Whilst it is recognised that there is potential for food processing to negatively impact the nutritional composition of foods it is also important to note that many food processes do not impact the nutritional quality of the food or can be beneficial (e.g. freezing of fresh vegetables to retain nutrients over time) (Lee et al., 2018; Favell, 1998).

A number of food classification systems exist to categorise foods based on degrees or types of food processing (de Araujo et al., 2022). Classification systems typically use terms like “highly” or “ultra-processed”, but overall, the generally accepted view of food processing among the food science community, is that food processing involves combining a series of procedures (unit operations) to achieve a “Specific, identifiable and predictable effect on a food” (Fellows, 2022). While this definition focuses on unit operations, which are physical in nature (e.g. heat processing, freezing, dehydration, evaporation, crystallisation etc), other classification systems are more focused on issues relating to product formulation (de Araujo et al., 2022) and not unit operations per se. These are then sometimes used to provide healthy eating advice, usually with recommendations to reduce foods in the highly “processed” categories, however these classification systems have also received criticism that is important to consider (Moradi et al., 2021a, 2021b; Suksatan et al., 2021; Delpino et al., 2022; Martini et al., 2021; Petrus et al., 2021; Gibney et al., 2017; Sadler et al., 2021; Knorr and Augustin, 2021). Indeed it is not uncommon to find processed foods with minimal amounts of added ingredients, and as such it will be important moving forward to align these differing approaches to defining processed foods (de Araujo et al., 2022; Fellows, 2022). Such terminology is also influencing consumer understanding of food processing, resulting in a negative association of food processing and health (Meijer et al., 2020).

Outside of the considerations of food processing, the impact of food production, food processing and consumption on the environment is also increasingly important to consider (United Nations, 2022). Production of food is inherently linked with the environment through land and water use as well as greenhouse gas emissions (Ritchie and Roser, 2022). Several metrics can be used to quantify the impact of foods on the environment including cropland use, water use, and greenhouse gas emissions associated with the production of the specific food or food group and combining all stages of the food lifecycle in a life cycle assessment can give an overarching picture of the impact of food(s) on the environment (Ritchie and Roser, 2022). Recent examination of diet quality and environmental impact of dietary intake data in the US highlighted the complexities when evaluating link between population and planetary health (O'Malley et al., 2023). In addition to understanding the link between diet quality and environmental impact, it is also important to consider how processing plays a role in this relationship. A recently published novel conceptual model highlighted the commercial, biological and social drivers of the processed food system, and the links to environmental sub-systems including climate, land, water and waste. This work highlighted the interactions between processing and environmental impact, demonstrating how changes to one component of the system could have flow-on effects on another (Anastasiou et al., 2022).

Nutrient composition, processing and sustainability are all important factors to consider in policy, food innovation and health promotion. Interconnections exist across all three and it is important to considered them together. Using model meal plans, this paper aims to examine the link between nutrient content, environmental impact, and processing.

## 2 Methods

**Fig. 1:**
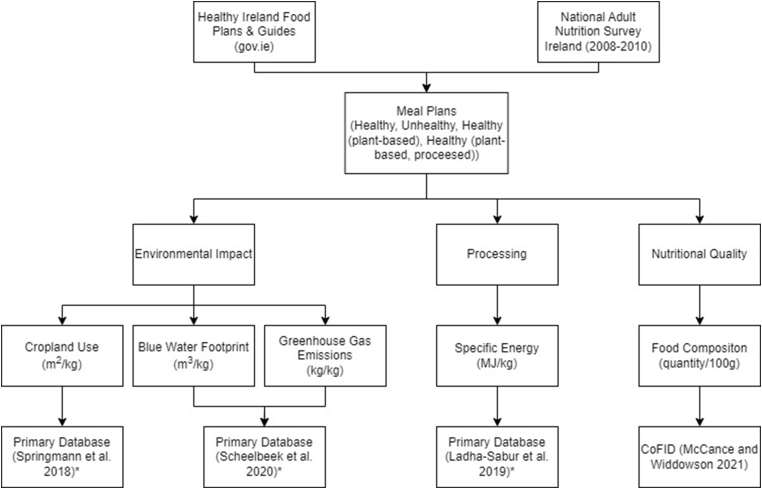
Outline of the framework and databases used to describe each metric. * indicates that this was the primary database used but values for some foods were obtained from other published sources where they were not available in the primary database. These alternative sources are outlined in the supplementary data file.

The approach used for this study is summarised in Fig. 1, which outlines the primary databases used for each metric (environmental impact, processing and nutritional quality). In summary, Healthy Ireland meal plans (Government of Ireland, 2022) were used along with data from the National Adult Nutrition Survey (NANS) (Irish Universities Nutrition Alliance, 2022) to determine four distinct meal plans – ‘Healthy’, ‘Unhealthy’, ‘Healthy (plant-based, processed)’ and ‘Healthy (plant-based)’. The rationale for devising these specific meal plans was to take into account current food based dietary guidelines (Healthy diet), as well as more recently recommendations which take into account environmental impact and focus on plant based diets (Government of Ireland, 2022) Within the plant based diets we considered an un/less-processed approach to this diet, and then a diet which includes processed plant foods (‘Healthy (plant-based)’ & Healthy (plant-based, processed) (Government of Ireland, 2022). These 3 diets are all considered healthy, devised to meet recommended nutrient intakes (Government of Ireland, 2022). Alongside these an ‘Unhealthy’ meal plan, representing a diet high in energy, saturated fat, and sodium, and low in fibre was also developed. For each of the meal plans the environmental impact, processing and nutritional composition metrics were compared (Fig. 1).

### 2.1 Meal plan selection

As described four model meal plans were designed to represent a typical ‘Healthy’ diet, an ‘Unhealthy’ diet, ‘Healthy (plant-based)’ & ‘Healthy (plant-based, processed)’ for an adult, >18yrs. Each diet consisted of a four day meal plan with each day's intake split into four meal types (breakfast, lunch, dinner, and snacks). The ‘Healthy’ diet was based on meal plans outlined in the Healthy Ireland Food Plans and Guides published by the government of Ireland (Government of Ireland, 2022). For the ‘Unhealthy’ diet, data from the NANS (Irish Universities Nutrition Alliance, 2022) was used to identify commonly consumed meals that were considered ‘unhealthy’ based on their nutritional content, i.e. were high in nutrients such as saturated fat and low in vitamins, minerals and fibre (World Health Organisation, 2020). The plant based diets were devised by substituting meat products present in the Healthy diet with non-meat food alternatives. When substituting meat products, both less “processed” plant foods (e.g. chickpeas, avocado) and more “processed” plant based alternatives (e.g. peanut butter, vegetable burger) were used for the ‘Healthy (plant-based)’ & ‘Healthy (plant-based, processed)’ diets, respectively. These were selected from foods commonly consumed in NANS and those available in local supermarkets (Irish Universities Nutrition Alliance, 2022). All food items used within the meal are listed in the supplementary data file. An example of one day's meal plan is given in Table 1 (full outlines of all meal plans available in Supplementary Material). Food portion sizes for all foods within each meal plan were determined using Food Portion Sizes (Ministry of Agriculture Fisheries and Food, 1994) and Carbs & Cals (Cheyette and Balolia, 2013).

**Table 1:** Example of a one day meal plan outlining foods used for each diet.

Meal Type	Healthy Plan	Unhealthy Plan	Healthy (plant-based, processed)	Healthy (plant-based)
**Breakfast**	Porridge with low-fat milk, raspberries and wholemeal/grain toast	White toast with butter, eggs, sausages, rashers, black pudding and tea with whole milk	Porridge with unsweetened soya milk, raspberries and wholemeal/grain toast	Porridge with unsweetened soya milk, raspberries and wholemeal/grain toast
**Lunch**	Egg, lettuce and tomato sandwich with low-fat yoghurt, oranges and water	Sandwich with butter and rashers, chocolate biscuits, sugar sweetened beverage (cola) and crisps	Vegetable burger sandwich with soya yoghurt and oranges	Tofu sandwich with soya yoghurt and oranges
**Dinner**	Pork and vegetable noodle stir fry and water	Roast beef, mashed potatoes, peas, carrots, tea with whole milk, and banoffee pie	Quorn pieces, carrots, mushrooms, and green beans stir fry with wholewheat noodles and water	Chickpea, carrot, green bean and mushroom stir fry with wholewheat noodles and water
**Mid-morning snack**	Pear and water	Milk chocolate and tea with whole milk	Pear and water	Pear and water
**Afternoon snack**	Apple	Crisps and chocolate	Apple	Apple
**Evening snack**	Wholegrain cereal with low-fat milk and banana	Chocolate biscuits	Wholegrain cereal with unsweetened soya milk and banana	Wholegrain cereal with unsweetened soya milk and banana

### 2.2 Nutritional composition

The nutritional composition of each meal plan was calculated using food composition data from Composition of Foods Integrated Dataset (CoFID) (UK Government, 2021). Nutritional composition of foods within each meal were determined using portion sizes for each food, and summed to give a total nutrient intake per day. Mean daily nutrient levels were calculated for each nutrient, for each diet based on the average of the four day plan to allow comparisons between the diets (Table 2, Table 3). The Alternate Healthy Eating Index (AHEI) was determined as a measure of diet quality for each meal plan (Table 4) (McCullough and Willett, 2006).

**Table 2:** Mean daily energy and nutrient composition of meal plans.

	Healthy		Unhealthy		Healthy (plant-based, processed)		Healthy (plant-based)	
Nutrient	Value	STDEV	Value	STDEV	Value	STDEV	Value	STDEV
**Energy (kcal/day)**	2466.2^a^	192.2	3701.0^b^	288.8	2109.4^a^	87.3	2099.1^a^	107.9
**Protein (g/day)**	171.2^a^	44.8	127.4^ab^	20.9	108.1^b^	8.5	81.9^b^	8.1
**Energy (kcal) from Protein (%/day)**	27.5^a^	5.8	13.8^b^	1.8	20.6^ab^	2.4	15.7^b^	2.1
**Fat (g/day)**	64.9^a^	16.0	196.0^b^	17.8	56.3^a^	22.4	57.6^a^	15.7
**Energy (kcal) from Fat (%/day)**	23.5^a^	4.6	47.7^b^	1.8	23.8^a^	8.5	24.6^a^	6.3
**Saturated fatty acids (g/day)**	25.0^a^	9.1	80.6^b^	12.6	11.5^a^	6.8	11.6^a^	3.9
**Energy (kcal) from Saturated Fat (%/day)**	9.1	3.3	19.6	3.1	4.9	2.9	5.0	1.7
**Polyunsaturated fatty acids (g/day)**	10.3^a^	1.9	22.4^a^	8.5	18.5^a^	7.0	16.8^a^	0.8
**Cholesterol (mg/day)**	519.9^a^	129.4	730.8^c^	135.4	4.0^b^	4.4	14.7^b^	19.0
**Carbohydrate (g/day)**	319.2^a^	46.7	380.4^a^	26.2	311.9^a^	27.7	334.2^a^	30.1
**Energy (kcal) from Carbohydrate (%/day)**	52.2^ab^	10.0	41.2^b^	1.2	59.3^a^	7.0	63.7^a^	5.0
**Starch (g/day)**	165.4^a^	35.9	221.4^a^	16.8	169.8^a^	35.1	196.5^a^	27.7
**Total sugars (g/day)**	153.1^a^	21.8	158.5^a^	30.6	136.6^a^	17.2	133.3^a^	20.4
**Non-starch polysaccharide (g/day)**	30.8^ab^	6.8	19.8^b^	4.0	36.5^a^	8.3	42.3^a^	5.7
**AOAC fibre (g/day)**	39.5^a^	8.0	19.1^b^	3.6	57.4^a^	14.2	58.3^a^	4.3

Differing superscript letters indicate statistically significant difference at p < 0.05 using ANOVA with post-hoc *t*-test with Bonferroni correction for multiple comparisons. Abbreviations; STDEV - standard deviation.

**Table 3:** Mean daily micronutrient composition (per 10 MJ) of the meal plans.

	Healthy		Unhealthy		Healthy (plant-based, processed)		Healthy (plant-based)	
Nutrient	Mean	STDEV	Mean	STDEV	Mean	STDEV	Mean	STDEV
**Sodium (mg/10 MJ)**	1827.4^a^	266.6	2822.5^c^	183.4	2290.9^a^	325.8	960.3^b^	375.4
**Calcium (mg/10 MJ)**	1794.5^a^	362.9	582.3^b^	62.4	1711.8^ab^	302.1	1627.6^ab^	322.9
**Vitamin D (μg/10 MJ)**	8.1^a^	7.4	3.1^a^	1.7	9.3^a^	2.3	9.4^a^	2.0
**Riboflavin (mg/10 MJ)**	3.7^a^	0.5	1.4^b^	0.1	3.9^a^	0.7	3.5^ab^	0.4
**Vitamin B12 (μg/10 MJ)**	9.5^a^	3.8	4.5^ab^	1.7	5.4^ab^	1.3	5.1^b^	1.0
**Vitamin C (mg/10 MJ)**	207.8^a^	39.4	31.2^b^	20.8	213.7^a^	59.8	223.4^a^	66.6

Differing superscript letters indicate statistically significant difference at p < 0.05 using ANOVA with post-hoc *t*-test with Bonferroni correction for multiple comparisons. Abbreviations; STDEV - standard deviation.

**Table 4:** Alternate Healthy Eating Index (AHEI), processing specific energy, greenhouse gas emissions, blue water footprint, cropland use, total number of processes in the diet, and average number of processes per food item in the diet values for each diet.

	Healthy		Unhealthy		Healthy (plant-based, processed)		Healthy (plant-based)	
	Mean	STDEV	Mean	STDEV	Mean	STDEV	Mean	STDEV
**AHEI Score**	61.61^a^	2.53	16.44^c^	5.44	69.94^ab^	11.20	78.96^b^	2.51
**Processing specific energy (MJ/day)**	4.93^a^	0.46	7.57^a^	0.76	17.33^b^	5.34	5.05^a^	0.32
**Greenhouse Gas Emissions (kg/day)**	5.32^ab^	0.72	5.57^b^	1.64	3.32^a^	0.38	3.29^a^	0.39
**Blue Water Footprint (m**^**3**^**/day)**	0.29^a^	0.14	0.18^a^	0.11	0.27^a^	0.18	0.22^a^	0.13
**Cropland Use (m**^**2**^**/day)**	6.00^a^	1.39	5.39^a^	1.19	11.62^b^	1.87	13.69^b^	2.12
**Total number processes in diet per day**	86.00^a^	13.39	131.25^b^	2.99	85.75^a^	10.53	86.50^a^	6.45
**Average number of processes per food item**	4.19^a^	0.23	5.59^b^	0.17	4.53^a^	0.30	4.30^a^	0.52
**Energy Density (kcal/g food)**	0.89^a^	0.09	2.24^b^	0.33	0.79^a^	0.08	0.75^a^	0.02

Differing superscript letters indicate statistically significant difference at p < 0.05 using ANOVA with post-hoc *t*-test with Bonferroni correction for multiple comparisons. Abbreviations; STDEV - standard deviation.

### 2.3 Environmental impact

Blue Water Footprint (m^3^/kg food) and Mean Greenhouse Gas Emissions (kg CO_2_ eq/kg food) data for each food item were extracted from a database published by Scheelbeek et al. (2020) (Scheelbeek et al., 2020). Cropland Use (m^2^/kg food) data was extracted from a database published by Springmann et al. (2018). In some cases an exact match for a food in the meal plan was not available. In these cases a closely related food product was used instead or the main ingredient of the food was used as a substitute. For example, where a value for pasta was not available the value used was for wheat. In a limited number of cases environmental impact data was obtained from an alternative published source. A full list of the assumptions made and alternative sources used for environmental impact data is available in Supplementary material. Like the nutritional quality, total values were obtained for each day, and mean diet values (average of the 4 days) were calculated (Table 4).

### 2.4 Processing metric

Values for processing specific energy (MJ/kg) consumption associated with the production of each food was used as a quantitative metric for processing. This data was mostly gathered from one published database (Ladha-Sabur et al., 2019), however for some foods not listed in this database values were gathered from alternative published articles which are referenced in Supplementary material. If no published processing specific energy value was available for a specific food, a value for a similar food or the main ingredient of the food was used as a substitute, for example soybeans instead of tempeh burger. Total processing specific energy values were obtained for each day, and mean diet values (average of the 4 days) were calculated (Table 4). These processing specific energy values are intended to represent the level of food processing with a higher value being associated with higher degree of food processing. In addition to using processing specific energy, processing was also considered by the number of unit operations (processes) required to manufacture a food (e.g. post-harvest/ingredient procurement and before packing/filling). A total number of processes for each diet was determined (sum of the 4 days). However, due to the variation in the number of individual food items in each diet, the number of processes was also divided by the number of food items in the diet to give a value for the average number of processes per food item.

### 2.5 Statistics

**Fig. 2:**
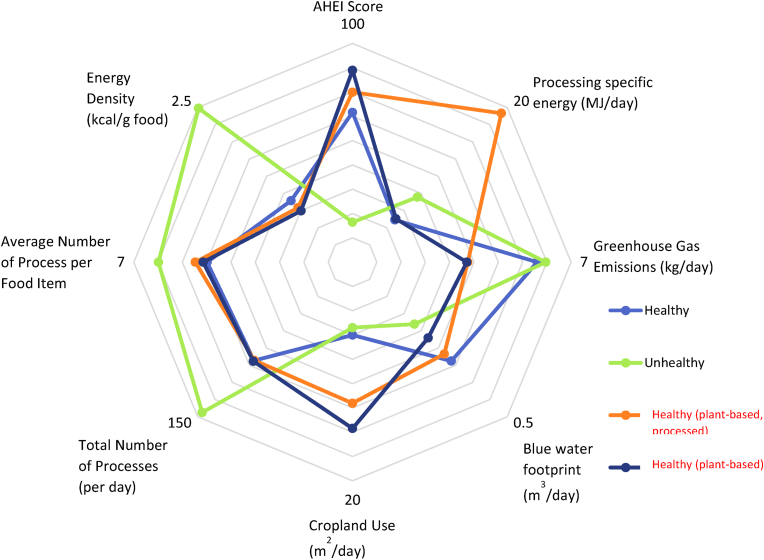
Radar plot displaying the aggregated data from four diets used in this study and their associated scores for the nutritional, processing, and environmental impact metrics.

Statistical analysis was conducted using R version 4.2.2 in the RStudio integrated development environment (version 2022.07.2 + 576). An ANOVA with post-hoc *t*-test with Bonferroni correction for multiple comparisons was applied with differences considered significant where p < 0.05. Pearson correlation plots were prepared to examine the interactions between nutrients, environmental and processing metrics with the r^2^ values being reported. A radar plot (Fig. 2) was prepared in Microsoft Excel to show the mean values obtained for each diet across all metrics including nutritional quality, processing, and environmental impact data.

## 3 Results

### 3.1 Nutritional quality and AHEI

Table 2 outlines the nutritional composition of the diets used in this study. Before comparison of key nutrients across the diets it is important to confirm the classification of the developed diets as ‘Healthy’ and ‘Unhealthy’. Only the ‘Unhealthy’ diet exceeded recommended intake of total fat (<30% total energy) and saturated fat (<10% total energy), with the ‘Unhealthy’ diet reaching 47% and 19% energy from total fat and saturated fat respectively. Recommended intake of fibre (25–34g/day) were only met by all diets except the ‘Unhealthy’ diet, with both plant based diets reporting in excess of 50g/day. Examining energy content, the ‘Unhealthy’ diet with 3701 kcal/day, provided significant more energy, than the other 3 diets, with respect to recommended energy intakes of 2000–2500 kcal/day.

Comparing nutrient intakes across the four diets, the ‘Unhealthy’ diet had significantly higher (p < 0.01) mean daily energy levels with 3701 kcal/day compared to ‘Healthy’ (2466 kcal/day), ‘Healthy (plant-based, processed)’ (2109 kcal/day) and ‘Healthy (plant-based)’ (2099 kcal/day) (Table 2). Average daily protein level in the ‘Healthy’ diet (171.2 g/day) was significantly greater than both of the plant based diets ('Healthy (plant-based, processed)’ 108.1 g/day and ‘Healthy (plant-based)’ 81.9 g/day) (p < 0.01) while the protein level in the ‘Unhealthy’ diet (127.5 g/day) was not significantly different to any of the other diets (Table 2). The average fat content was similar across the ‘Healthy’, ‘Healthy (plant-based, processed)’ & ‘Healthy (plant-based)’ diets with values of 64.9 g/day, 56.3 g/day and 57.6 g/day, respectively while the ‘Unhealthy’ diet had a significantly higher (p < 0.01) average fat content with 196.0 g/day (Table 2). Mean daily saturated fat content was similar for the ‘Healthy (plant-based, processed)’, ‘Healthy (plant-based)’ and ‘Healthy’ diets with values of 11.5 g/day, 11.6 g/day and 25 g/day, respectively, while the ‘Unhealthy’ diet had a significantly higher (p < 0.01) level of 80.6 g/day (Table 2). Cholesterol levels for the two plant based diets were significantly lower (p < 0.01) than the other diets with 4.0 mg/day for the ‘Healthy (plant-based, processed)’ and 14.7 mg/day for the ‘Healthy (plant-based)’ diet. The ‘Unhealthy’ diet had the highest (p < 0.01) level with 730.8 mg/day while the ‘Healthy’ diet had 519.9 mg/day which was greater than the plant based diets and less than the Unhealthy diet (p < 0.01) (Table 2). Total sugars in the diet did not vary significantly (p > 0.05) between the four diets with values ranging from 133.3 to 158.6 g/day (Table 2). The ‘Unhealthy’ diet had a significantly lower (p < 0.01) fibre level (19.1 g/day) compared to the two plant based diets which had values of 57.4 g/day and 58.3 g/day for the ‘Healthy (plant-based, processed)’ and ‘Healthy (plant-based)’ diets, respectively and the ‘Healthy’ diet which had 39.5 g/day of fibre (Table 2).

Table 3 shows the mean daily nutrient composition per 10 MJ for each of the meal plans. Mean daily sodium content was highest (p < 0.01) for the ‘Unhealthy’ diet (2822.5 mg/10 MJ) with the ‘Healthy (plant-based, processed)’ diet and ‘Healthy’ diet having the next highest levels and being similar (p < 0.01) to each other with levels of 2290.9 mg/10 MJ and 1827.4 mg/10 MJ, respectively. The ‘Healthy (plant-based)’ diet had the lowest (p < 0.01) sodium level with 960.3 mg/10 MJ (Table 3). The only statistical difference (p < 0.01) in calcium levels was between the ‘Healthy’ and ‘Unhealthy’ diets which had values of 1794.5 mg/10 MJ and 582.3 mg/10 MJ, respectively (Table 3). Vitamin D levels were similar (p > 0.01) across the four diets with values ranging from 3.1 to 9.4 μg/10 MJ (Table 3). Vitamin C levels were significantly lower (p < 0.01) for the ‘Unhealthy’ diet (31.21 mg/10 MJ) compared to the other three diets which had values ranging from 207.78 to 223.43 mg/10 MJ (Table 3).

The AHEI scores for each of the diets is presented in Table 4 and visualised as part of Fig. 2. As expected, the AHEI for the ‘Unhealthy’ diet (16.44) was significantly lower (p < 0.01) than the other three diets (Table 4). The ‘Healthy (plant-based)’ diet had the highest (p < 0.01) AHEI with a score of 78.96 which was similar to the value for the ‘Healthy (plant-based, processed)’ diet of 69.94 (Table 4).

### 3.2 Specific energy of processing

The ‘Healthy’, ‘Unhealthy’, and ‘Healthy (plant-based) diets had similar (p > 0.05) processing specific energy consumption with values of 4.93 MJ/day, 7.57 MJ/day, and 5.05 MJ/day, respectively (Table 4). The ‘Healthy (plant-based, processed) diet had significantly greater (p < 0.01) specific energy consumption at 17.33 MJ/day (Table 4). The ‘Healthy’, ‘Healthy (plant-based, processed)’ and ‘Healthy (plant-based)’ diets all had a similar (p > 0.05) number of processes associated with them (85.75–86.50) while the ‘Unhealthy’ diet had a significantly greater (p < 0.01) number of associated processes with n = 131.25 (Table 4). When the number of food items in each diet is taken into consideration the ‘Unhealthy’ diet still had a significantly higher (p < 0.01) number of processes compared to the other three diets (Table 4). The energy density (kcal/g food) was significantly higher (p < 0.01) for the ‘Unhealthy’ diet compared to the other three diets (Table 4).

### 3.3 Environmental impact

Greenhouse gas emissions were similar for the ‘Healthy’ and ‘Unhealthy’ diets with total values of 5.32 kg/day and 5.57 kg/day, respectively (Table 4). The greenhouse gas emissions for the two plant based diets were significantly lower (p < 0.01) than the ‘Unhealthy’ diet (Table 4). Blue water footprint was similar between the four diets (Table 4). Cropland use was similar (p > 0.05) for the ‘Healthy’ and ‘Unhealthy’ diet with total values of 6.00 m^2^/day and 5.39 m^2^/day, respectively (Table 4). The cropland use for the two plant based diets were similar to each other but significantly higher (p < 0.01) than the ‘Healthy’ and ‘Unhealthy’ diets with values of 11.62 m^2^/day and 13.69 m^2^/day, for ‘Healthy (plant-based, processed)’ and ‘Healthy (plant-based)’ respectively (Table 4).

### 3.4 Diet comparisons

Fig. 2 outlines the nutritional quality, environmental impact and processing data for each of the diets used in this study. This visual representation allows for quick and clear comparisons to be made between the diets for the various metrics studied.

### 3.5 Nutrient, processing & environmental metric correlations

Pearson correlation analysis was completed to investigate the relationship between the processing specific energy and number of processes per food associated with an individual food and the nutrient composition of that food (Table 5). No clear correlations between the processing specific energy consumption associated with production of each food and nutrient values was evident. However, strong positive correlations were evident when the number of food processes in the diet and composition of nutrients was examined. In particular, energy density (r^2^ 0.832), sodium (r^2^ 0.737) content, saturated fat (r^2^ 0.852) and total energy (r^2^ 0.808) (Table 5).

**Table 5:** Correlation coefficient values of processing specific energy values (MJ/100g) or estimated number of processes in the diet and a range of nutrients.

Nutrient	Processing specific energy r^2^	Number of processes r^2^
**Total sugars (g/100g)**	0.009	0.004
**Saturated fatty acids (g/100g)**	0.054	**0.852**
**Sodium (mg/100g)**	0.011	**0.737**
**Cholesterol (mg/100g)**	0.140	0.407
**Calcium (mg/100g)**	0.001	0.436
**Vitamin D (μg/100g)**	0.027	0.302
**Riboflavin (mg/100g)**	0.039	**0.695**
**Vitamin B12 (μg/100g)**	0.054	0.002
**Vitamin C (mg/100g)**	0.000	0.585
**Energy (kcal/100g)**	0.051	**0.808**
**Energy Density (kcal/g)**	0.025	**0.832**

Determination coefficients represented by r values for the model diets derived using Pearson correlations.

Values highlighted in bold represent a significant correlation.

Similar analysis was completed to determine the relationship between the processing specific energy and number of processes per food associated with an environmental metrics of that food (Table 6). No clear correlations between the processing specific energy consumption associated with specific energy for each food and environmental impact was evident. When considering the number of food processes in the diet there was a significant correlation between the number of processes per food and cropland use (r^2^ 0.364) (Table 6).

**Table 6:** Correlation coefficient values of processing specific energy values (MJ/100g) or estimated number of processes in the diet and greenhouse gas emissions, blue water footprint & cropland use.

Nutrient	Processing specific energy r^2^	Number of processes per food item r^2^
**Number of processes per food item**	0.113	1.00
**Mean greenhouse gas emissions**	−0.021	0.167
**Blue Water Footprint**	−0.065	0.044
**Cropland Use**	−0.027	**0.364**

Determination coefficients represented by r values for the model diets derived using Pearson correlations.

Values highlighted in bold represent a significant correlation.

## 4 Discussion

This study sought to determine the relationship between food processing, diet quality and environmental impact in a range of model diets. Across the model diets considered in this study, there was no clear link between diet quality and food processing when considered using processing specific energy value. However, when the number of processes in each diet was estimated, the unhealthier diet had considerably more processes associated with it. Similarly, there was no clear link between specific energy associated with the production of food and environmental metrics. Examining the interaction of nutritional quality, environmental impact and processing of foods and diets in this novel way highlights the complexity of this issue.

The model diets selected aimed to consider healthy dietary practices, including plant based or plant rich approaches to healthy eating which are being recommended, and unhealthy dietary practices, based on current intakes within existing national surveys. Across these diets, nutritional quality, environmental impact and processing were deliberately considered in a number of different ways. Applying the AHEI, which takes into account a range of dietary factors including but not limited to intake of fruit and vegetables, sugar-sweetened beverages and sodium, allowed the diets to be considered in totality rather than focusing on a single nutrient. In doing so, the current paper found no clear link between the overall healthfulness of the diet and the energy associated with its production/processing. The link between processing and diet quality was evident but depended on the metric considered. When the number of processes per food was considered the ‘Unhealthy’ diet did have the highest total number and average number of processes per food. However, the total specific energy associated with the production of the diets was considered, there was no link to nutrient quality or healthfulness of the diet, in contrast to much of the existing literature.

Much of the existing literature to date has focused on the links between processed food, nutrient intake and health using diets classified using the NOVA food system amongst others. When we look at the studies together in a meta-analysis Moradi et al. (2021a) has demonstrated a clear relationship between UPF intake and risk of obesity. Within an RCT examining the link between processed food intake and health, Hall et al. (2019) investigated whether consumption of “ultra-processed” foods affected energy intake, finding that ∼500 more kcal/day was consumed on a diet of “ultra-processed” foods versus an unprocessed diet, suggesting that limiting consumption of “ultra-processed” food may be effective in preventing and treating obesity (Hall et al., 2019). Whilst this seems a causal link between processing and health/disease, it is important to note that by definition the foods in the UPF category are higher in energy, fat, and sugar – nutrients that we know are associated with increased risk of obesity and other metabolic disorders (Moradi et al., 2021a, 2021b; Suksatan et al., 2021; Delpino et al., 2022). Whether processing or nutrients are responsible for this is a question that we need to answer to properly frame/contextualise the issue of processed foods and health.

Further examining nutrient quality, in the present study, higher energy density was observed for the ‘Unhealthy’ diet compared to the other three model diets. Higher energy density is known to influence ad libitum energy intake and, and has recently been considered alongside eating rate to investigate energy intake rate (kcal/min) (Forde et al., 2020). This link between energy density and processing is not new. Forde, Mars and de Graaf (2020) concluded that energy intakes rates should be considered when comparing unprocessed and “ultra-processed” diets in relation to “ultra-processed” food consumption and obesity (Forde et al., 2020). The authors noted “ultra-processed” foods, on average, have a faster energy intake rate (kcal/min) compared to unprocessed foods which can contribute increased energy intake and body weight (Forde et al., 2020). A strategy that has been proposed to reduce energy intake rates associated with “ultra-processed” foods is through altering food texture to reduce eating rate (Teo et al., 2022). Meals with a “hard” texture were consumed slower overall with a reduced energy (kcal) consumption compared to “soft” meals which had higher intakes (Teo et al., 2022).

Unlike the traditional approach where foods are categorised into specific processing categories, this paper aimed to take a more quantitative approach, quantifying processing by considering the amount of energy and the number of processes involved in the production of each food item in the model diets contained. In completing this analysis it was evident that, even when considering simple model diets, as examined here, there is a lack of data on the specific energy associated with processing of foods, and data that is available can vary greatly depending on location, scale, time of the study and efficiency of equipment used (Ladha-Sabur et al., 2019). Much of the data focuses on industrial processing and does not take into account processing within the home. A database with processing specific energy data for manufacturing and distribution of foods published by Ladha-Sabur et al. (2019) (Ladha-Sabur et al., 2019) was used in this study as the primary dataset. Whilst this is a unique and valuable resource, this database includes processing specific energy values from a range of sources collected across a number of years, meaning that there is potential for discrepancies/inaccuracies in the data due to variation between studies, changes in energy efficiency and use of different technologies amongst others (Ladha-Sabur et al., 2019). In addition, some of the energy values presented included energy required for harvesting, packaging, storage or distribution while others did not (Ladha-Sabur et al., 2019). As the database did not provide a breakdown of the value for all foods this is a source of possible error. Furthermore, it did not take intake account the additional energy costs associated with processing (heating) within the home, which is known to vary significantly (Sonesson et al., 2005; Arrieta and González, 2019). Similarly, estimating the number of processes used to make a food and sub-categorising them based on the type of unit operation (heating, cooling, mixing, cutting, etc.) may be useful but again requires substantial work to be a viable system. It is important that such datasets are further developed expanding both the information about the existing foods as well as the number of food listed. In addition, limits should be defined for where food processing begins and ends. For example, should energy required to harvest wheat be included in the final processing specific energy for pasta manufacturing or should the energy needed to pack the final product or store it prior to distribution be included? Comprehensive datasets on the nutrient composition of food have existed for some time, and are used widely in food and nutrition research in examining the link between nutrition and health (UK Government, 2021). Lack of data about processing of foods limits the ability to truly investigate purported links to nutrient intake and health.

These findings on the environmental impact of the diets examined here are similar to O’Malley et al. (2023), who examined carbon footprint and diet quality of popular diets in the US. Similar to the findings here, the average carbon footprints of vegan and vegetarian diets were lower than those of the pescatarian, omnivore, paleo or keto diets (O'Malley et al., 2023). However this study did not examine multiple elements of environmental impact, which as demonstrated within our study vary significantly. Examining the environmental impact of the diets within this paper, three metrics were chosen; blue water footprint, cropland use and greenhouse gas emissions. The blue water footprint did not vary greatly between the 4 diets while the greenhouse gas emissions were higher for both the Healthy and Unhealthy diet compared to the two plant based diets. The greater emissions for non-plant based diets was attributed mostly to the higher emissions associated with the meat products included in the diets. The cropland use was significantly greater for both of the plant based diets compared to the Healthy and Unhealthy diets. The cropland use metric will naturally skew towards a higher value for plant based diets and is worth considering when reviewing the data.

Considering the current trend towards more sustainable plant based diets it is important to consider the effects this will have across the food system (Mertens et al., 2016; Willett et al., 2019). Recommendations to reduce meat consumption to reduce environmental impact may lead people to consume more highly “processed” plant based alternatives. As seen in this study the number of processes associated with the ‘Healthy’ diet (meat based) and both plant based diets was similar. With this in mind it is crucial that when considering diet quality and making recommendations for the future, all aspects of the food system are considered. If a shift towards a more sustainable plant based diet leads to an increase in the consumption of “processed” foods then there is potential for a contradiction between recommendations to occur. This point is highlighted by Anastatiou et al., where a series of models conceptualising the drivers of ultra-processed food production and consumption and their environmental impacts highlighted the complex links between the commercial, biological and social drivers of the UPF system, and the impacts on environmental sub-systems including climate, land, water and waste. It notes certain trade-offs on various parameters that need to be considered when developing sustainable food policies including food waste and food safety (Anastasiou et al., 2022).

## 5 Limitations

As discussed in the manuscript, significant data gaps were present for some of the metrics used in the study and resulted in the need for a number of assumptions to be made. However, this manuscript acts as a proof of concept to both demonstrate the potential of examining food systems across multiple metrics at once and also highlights where data gaps exist currently to allow them to be filled over time. Rather than using a categorical approach, food processing was considered in more novel ways within this study, using data for processing specific energy consumption associated with food production and the estimated number of processes required to manufacture a food. The authors are not saying these are the definitive approaches to be used, and there are certainly alternative approaches to quantify food processing, however, the current study is a useful starting point to open discussions to move toward a quantitative approach to examine food processing. Finally, this study examined a limited number of diets and chose to use a model diet approach rather than looking at actual intake data from a population. Work presented in this paper considered model diets, ranging from recommended intakes, including those incorporating sustainability (Government of Ireland, 2022), to current unhealthy eating patterns, typical for the Irish population and derived from existing food consumption surveys. This approach allowed us to consider how to obtain an combine data across domains of nutrient quality, processing and sustainability. Future studies should consider the use of actual intake data from large populations and expand the scope by linking to health parameters.

## 6 Conclusion

This study aimed to examine the interactions between diet quality, environmental impact and food processing in a range of model diets. This study highlights the number of different ways processing within a diet can be considered, using qualitative approaches such as specific energy and number of processes applied in production. Whilst comprehensive data exists for other parameters in food, this work highlights a clear gap in data of food processing which needs to be addressed as matter of priority. By incorporating metrics for diet quality, environmental impact and processing it will be possible to gain a clear insight into the impact a food has on both the global population and planet to improve dietary recommendations for the future.

## CRediT authorship contribution statement

**Steven L. Mulrooney:** All authors contributed to the development of the research, completed the, Formal analysis, Writing – original draft. **James G. Lyng:** All authors contributed to the development of the research, completed the, Formal analysis. **Cathal O'Hara:** All authors contributed to the development of the research, completed the, Formal analysis. **Aifric O'Sullivan:** All authors contributed to the development of the research. **E. Dolores O'Riordan:** All authors contributed to the development of the research. **Eileen R. Gibney:** All authors contributed to the development of the research, completed the, Formal analysis, Writing – original draft, All authors edited and approved the manuscript.

## Declaration of competing interest

The authors declare the declarations of interest: Author Eileen R. Gibney has received grant funding for research, but has no conflict of interest. Eileen R. Gibney has received research funding through the Food for Health Ireland project (Enterprise Ireland), Science Foundation Ireland Insight Centre for Data Analytics, and Horizon Europe (FNSCloud, PLANEAT), as well as research funding from Marigot Ltd, Ireland, and Société des Produits Nestlé, Switzerland. The funders listed were not involved in any of the work presented in this paper. Eileen R. Gibney has no other relationships or activities that have influenced the presented work. No other authors report declarations of interest.

## Data Availability

Data will be made available on request.
